# Description of the Buildings

**Published:** 1914-07-04

**Authors:** 


### Description of the Buildings.

(From a Correspondent.)

The admission department is immediately to the left on entering the main gateway. It consists of waiting room, with telephone room and janitor's quarters adjoining, and four examination rooms, each containing the necessary materials for record taking. The admission bathing-room is of special design to allow of the thorough cleansing of the patient and subsequent disinfection of clothes without risk of introducing contamination of any kind, as there is in connection with this room a "Reck" disinfector. This is quite irrespective of the large disinfector at the washing house. A small clinical laboratory, with incubator, etc., is included in this department, where immediate examination may be made of any material before the patient is admitted. All examination rooms are tiled to the roof, so that these can be thoroughly cleaned, and, when necessary, efficiently disinfected.

(Continued on page 385.)

### New Royal Hospital for Sick Children, Glasgow.

### Description of the Buildings (Continued from page 380).

To the west of this block, and connected thereto by the main corridor, running east and west, is the central block B, adjoining which is the general kitchen fitted with the most modern cooking appliances, every item being arranged to facilitate rapid working and thorough cleansing. Labour-saving devices—*e.g.*, electric mincing machines, as well as a potato peeler—are installed. All the hot air from the hot plate, steamers, and boiling coppers is extracted by a fan, which keeps the kitchen at a comfortable working temperature. Ample larder accommodation is provided, and there is a convenient and separate entrance for stores. A refrigerator is used for milk storage, and sterilisation will be done in the adjoining room by special apparatus.

On the main floor above are the dining-rooms for the resident and nursing staff, each being served by a pantry, which communicates with the kitchen corridor by a push-button lift.

The two higher storeys are fitted as maid's bedrooms, and there is a recreation-room for their own use, in addition to a dining-room which adjoins the kitchen corridor.

To the north of this block a conservatory leads to the administrative building, which forms the principal entrance to the institution for visitors and doctors. This building is four storeys in height, with basement. On entering, there is a large vestibule—to the left the board-room and matron's quarters are placed, and on the right the entrance to the Nurses' Home, which is entirely shut off from the general hospital. It includes, in the basement store-rooms, work-rooms, and nurses' box-room, with accommodation for the Ladies' Dorcas Society; on the ground floor, sisters' rooms, nurses' drawing and writing rooms, visitors' room, and the assistant matron's quarters; while on the upper flats, which are three in number, are the bedrooms for the staff. Each bedroom has a large cupboard (in place of the limited accommodation of a wardrobe), which is separately ventilated from the room and communicates with the extraction shaft.

The board-room is fitted with special accommodation for the records of the hospital. These will be kept in panel oak bookcases, and by means of a card system, embodying the name and the disease, any patient's record can be found within three minutes.

Returning to the main corridor, to the left is the dietetic kitchen, also a room for lecturing and giving practical demonstrations to nurses, and at the end the residents' quarters, which accommodate six residents. They are spacious and afford each man a sitting-room and bedroom, with the usual lavatory accommodation. This block is situated at the extreme eastern end of the hospital, and has ready access to the wards and admission department, which it adjoins. To the right is the operating-theatre block, having as a special feature the artificial lighting of each theatre by a searchlight placed outside, from which the light passing through a glass screen is reflected on to the operating area by means of mirrors, and by flush wall-lamps, which are watertight, allowing the whole of the walls to be hosed down, thus obviating the necessity of pendants or brackets of any kind, which cannot fail to collect dust. Students are admitted to either theatre by a side entrance, so that they never come in direct contact with the nurses or operator. Between the two operating theatres are two rooms, one for sterilisers and the other to be used as a nurses' robing-room. The sterilisation of dressings is done centrally in a large room directly under the operating theatre. There is also a lecture-room and $x$-ray department fitted up with the latest apparatus.

Abutting off the main corridor are the ward units, ten in number, self-contained in every respect. They accommodate sixteen to eighteen beds (with the exception of the central block C, which has arrangements for nurslings), with a special isolation block attached. This consists of three rooms, containing in all six beds. Supervision is easy from the corridor, as the partitions are principally plate-glass. In addition, each ward has attached the sanitary annexe, which has a special and separate ventilation system, and is provided with complete sanitary fittings, baths, hot-closets for towels, bed-pan washer, bed-pan steriliser, and a private lavatory for nurses.

Hand-basins are provided, and the bath has a patent top which makes it impossible to scald the child, as cold water must always be turned on first. There is a sink-room for the ward-maid, and a linen-room specially ventilated, with sparred cupboards on wheels. These have sloping tops to prevent the accumulation of dust.

A sister's duty-room is provided, and a test-room, where all the usual clinical methods can be carried out. In this there is a fume cupboard, arranged so that the ventilation is entirely separate from the rest of the hospital, and here can be kept any specimens necessary until the physician's visit. A surgical-dressing room is also provided with hot and cold sterile-water apparatus, instrument-steriliser, and hot chamber for towels.

The ward kitchen contains a cupboard for hot plates, push-button lift communicating with the kitchen corridor, and specially designed gas stove. A children's play-room, the occupants of which can be overseen from the ward (as the partition is all glass instead of being solid), is furnished with suitable toys, small tables, chairs, and cupboard.

The wards have large windows, double glazed, with low sills, so that the children can see out, the radiator being placed in the butts and thoroughly protected by means of iron guards. The air for ventilation passes through a light copper screen below the radiator, which can be moved easily and thoroughly cleaned.

Block C, to the south, in the centre row of buildings, is specially designed for the treatment of infants. Accommodation is provided so that mothers may be admitted in cases where this is necessary. Some of the features of the ward are unique, and worthy of the careful consideration of those specially interested in the treatment of nurslings and young children.

The sanitary arrangements of the building differ entirely from the usual sanitary tower, which necessitates the carrying of soil-water and other pipes to the extreme back of every ward pavilion. In this hospital all pipes are concentrated at one part in the central shaft, and all joints are exposed, so that the workmen can see at once any defect, and there is no possibility of sewer-water accumulation or any chance of contamination around the pavilion.

All the roofs are flat, and not only can they be made use of as play-rooms, but each roof has complete sanitary arrangements, as well as a ward kitchen. The elevators for patients and for food go from the basement to the roofs, which will form delightful roof-gardens. The balconies are very spacious, and could accommodate half the number of patients in the wards.

